# Crystal structure and Hirshfeld surface analysis of 6-methyl-2,3-di­hydro-1,3-benzo­thia­zol-2-iminium 3-(1,3-benzo­thia­zol-2-ylsulfan­yl)propanoate

**DOI:** 10.1107/S2056989026010686

**Published:** 2026-10-20

**Authors:** Abduvali Khushbokov, Khayit Turaev, Bekmurod Alimnazarov, Ibragim Umbarov, Khurshid Tursunov, Odil Choriyev, Jamshid Mengnorovich Ashurov

**Affiliations:** a Termez State University, Barkamol Avlod Str. 43, Termez 190111, Uzbekistan; b Institute of Bioorganic Chemistry, Academy of Sciences of Uzbekistan, 100125, M. Ulugbek Str 83, Tashkent, Uzbekistan; Vienna University of Technology, Austria

**Keywords:** crystal structure, organic salt, benzo­thia­zol-3-ium, proton transfer, charge-assisted hydrogen bonding

## Abstract

In the title salt, C_8_H_9_N_2_S^+^·C_10_H_8_NO_2_S_2_^−^, formed by proton transfer from the carb­oxy­lic acid to the ring N atom of 6-methyl-1,3-benzo­thia­zol-2-amine, a 6-methyl-2,3-di­hydro-1,3-benzo­thia­zol-2-iminium cation (*A*) and a 3-(1,3-benzo­thia­zol-2-ylsulfan­yl)propano­ate anion (*B*) are present. Deprotonation of the carboxyl group is evidenced by its two distinctly different C—O bond lengths of 1.281 (6) and 1.220 (6) Å, while protonation of the ring N atom is confirmed by the localization of the corresponding H atom in a difference-Fourier map. Despite their different ionic states, the benzo­thia­zole fragments of the two components retain closely comparable bond lengths and angles. The cation and anion are linked by three charge-assisted N—H⋯O hydrogen bonds. Two of these, involving the ring N—H group and one of the amino N—H groups, bridge the two carboxyl­ate O atoms of a single anion to form an *R*^2^_2_(8) graph-set motif; the third links these ion pairs into layers parallel to (100). Hirshfeld surface analysis, performed separately for components *A* and *B*, shows that H⋯H contacts dominate the surface for *A* (38.9%), followed by C⋯H/H⋯C (24.2%), O⋯H/H⋯O (13.0%) and S⋯H/H⋯S (11.9%) contacts. For anion *B*, the principal contributions are H⋯H (27.5%), S⋯H/H⋯S (25.7%), C⋯H/H⋯C (17.2%) and O⋯H/H⋯O (15.3%). The complementary H⋯O/O⋯H contributions are consistent with the important role of the charge-assisted N—H⋯O hydrogen bonds in consolidating the crystal packing.

## Abstract 2

In the title salt, proton transfer from the carb­oxy­lic acid to the benzo­thia­zole ring N atom generates a 6-methyl-2,3-di­hydro-1,3-benzo­thia­zol-2-iminium cation and a 3-(1,3-benzo­thia­zol-2-ylsulfan­yl)propano­ate anion. The ions are linked by N—H⋯O hydrogen bonds into *R*^2^_2_(8) motifs, which are further connected into layers parallel to (100).

## 1. Chemical context

Benzo­thia­zole is an aromatic heterobicyclic scaffold formed by fusion of a benzene ring with a thia­zole ring, combining an endocyclic N atom and an S atom within a single rigid framework (Padilla-Martínez *et al.*, 2025 ▸). This dual-heteroatom arrangement gives the core exceptional versatility as a hydrogen-bonding acceptor and as a metal-coordinating site, while substitution at the 2-position allows fine control over its donor⋯acceptor character, making benzo­thia­zole an extensively exploited heterocycle in medicinal and supra­molecular chemistry (Yadav *et al.*, 2023 ▸). Benzo­thia­zole derivatives display a remarkably broad spectrum of biological activities, including anti­microbial, anti­tumour, anti-inflammatory, anti­diabetic, anti­viral and anti-Alzheimer’s properties (Yadav *et al.*, 2023 ▸; Al-Mutairi, 2023 ▸), and several of these compounds are established clinical agents, among them riluzole (amyotrophic lateral sclerosis), ethoxzolamide (glaucoma), frentizole (immunosuppressive) and the amyloid-imaging probe thio­flavin-T (Padilla-Martínez *et al.*, 2025 ▸). Beyond medicinal chemistry, benzo­thia­zole derivatives are of considerable practical importance as rubber-vulcanization accelerators, corrosion inhibitors, dyes and agrochemical inter­mediates.
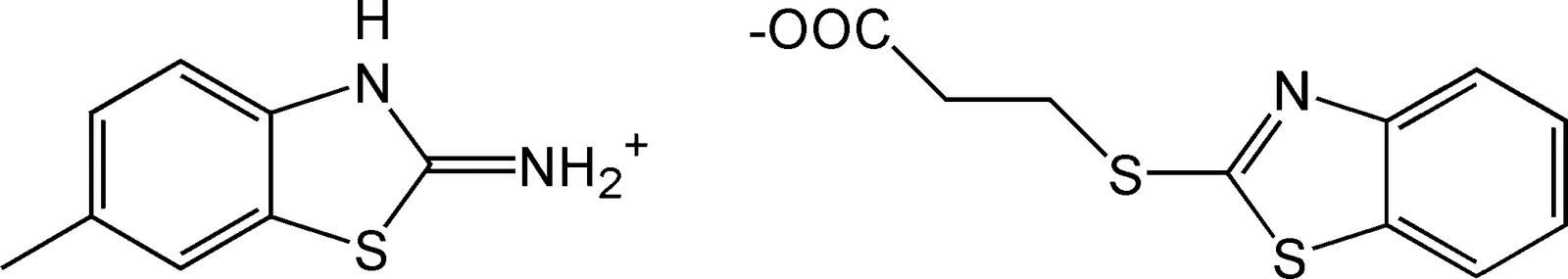


In 2-amino­benzo­thia­zoles, the endocyclic N atom can act as a proton acceptor, while the exocyclic amino group provides additional hydrogen-bonding donor sites. Conversely, S-functionalized derivatives of 2-mercaptobenzo­thia­zole combine a polarizable thio­ether linkage with other functional groups such as carb­oxy­lic acids.

Salts formed by proton-transfer between carb­oxy­lic acids and heterocyclic bases are particularly useful for crystal-engineering studies because proton transfer creates complementary charged donor and acceptor sites. The resulting N—H^+^ and COO^−^ groups commonly generate short, charge-assisted N—H⋯O hydrogen bonds and robust supra­molecular synthons. Previous structural studies of 2-amino­benzo­thia­zole with carb­oxy­lic acids have shown that protonation commonly occurs at the endocyclic N atom and that the ionic components are frequently organized by paired N—H⋯O inter­actions (Lynch *et al.*, 1998 ▸, 1999 ▸; Zhang *et al.*, 2009 ▸; Ilkimen *et al.*, 2013 ▸).

Synthesis and crystal structure determination of benzo­thia­zole-derived carb­oxy­lic acids and their salts have also been a continuing subject of our work (Ashurov, 2016 ▸; Ashurov *et al.*, 2016 ▸, 2017 ▸, 2018 ▸). Herein, we report the crystal structure and Hirshfeld surface analysis of a new proton-transfer salt formed between 3-(1,3-benzo­thia­zol-2-ylsulfan­yl)propanoic acid and 6-methyl-1,3-benzo­thia­zol-2-amine. Single-crystal X-ray diffraction demonstrates complete proton transfer from the carboxyl group to the ring N atom of the amino­benzo­thia­zole component, giving the 1:1 organic salt C_8_H_9_N_2_S^+^·C_10_H_8_NO_2_S_2_^−^.

## 2. Structural commentary

The asymmetric unit of the title salt contains one 6-methyl-2,3-di­hydro-1,3-benzo­thia­zol-2-iminium cation, (*A*, with all atoms of this unit designated by suffix *A*) and one 3-(1,3-benzo­thia­zol-2-ylsulfan­yl)propano­ate anion (*B*, with all atoms of this unit designated by suffix *B*), Fig. 1 ▸. The compound crystallizes in the ortho­rhom­bic space group *Pna*2_1_ with *Z* = 4.

Proton transfer was clearly established by the location of atom H1*A* at the endocyclic atom N1*A* in a difference-Fourier map; the exocyclic amino atom N2*A* carries two H atoms. Within the protonated 2-amino­benzo­thia­zolium fragment, the N1*A*—C1*A* and N2*A*—C1*A* distances are 1.316 (6) and 1.300 (7) Å, and the N2*A*—C1*A*—N1*A* angle is 124.8 (5)°. The near-equality of the two N—C1*A* bond lengths indicates substantial delocalization of the positive charge over the N1*A*—C1*A*—N2*A* fragment, a feature typical of protonated amidine-like systems. The cation is essentially planar: all non-H atoms lie within 0.013 Å of its least-squares plane.

The anion contains a benzo­thia­zole ring system attached through the exocyclic sulfanyl atom S2*B* to a propano­ate chain. The N1*B*—C1*B*, S1*B*—C1*B* and S2*B*—C1*B* bond lengths are 1.298 (7), 1.745 (5) and 1.747 (6) Å, respectively, while S2*B*—C8*B* is 1.809 (5) Å. The benzo­thia­zole portion of the anion is also essentially planar; inclusion of S2*B* gives a maximum deviation of 0.037 Å from the least-squares plane, whereas the propano­ate chain is markedly displaced from this plane.

The mean planes of the benzo­thia­zolium ring system of the cation and the benzo­thia­zole ring system of the hydrogen-bonded anion within the asymmetric unit subtend a dihedral angle of 56.06 (17)°.

The carboxyl­ate group is characterized by C10*B*—O1*B* and C10*B*—O2*B* distances of 1.281 (6) and 1.220 (6) Å, respectively, and an O2*B*—C10*B*—O1*B* angle of 124.2 (4)°. The unequal C—O distances reflect the different hydrogen-bonding environments of O1*B* and O2*B* (Table 1 ▸): O1*B*, which is involved in the longer C—O bond, accepts two N—H⋯O hydrogen bonds, whereas O2*B* accepts only one.

The flexible sulfanyl­propano­ate substituent adopts a folded conformation relative to the benzo­thia­zole plane. The C1*B*—S2*B*—C8*B*—C9*B* torsion angle is −73.6 (4)°, whereas the terminal part of the chain is close to *anti*, with S2*B*—C8*B*—C9*B*—C10*B* = −174.5 (3)°. The carboxyl­ate group is nearly coplanar with the C8*B*—C9*B*—C10*B* segment [C8*B*—C9*B*—C10*B*—O1*B* = −175.4 (4)° and C8*B*—C9*B*—C10*B*—O2*B* = 4.1 (6)°]. The conformation of the chain is accompanied by an intra­molecular C9*B*—H9*BA*⋯N1*B* contact (Table 1 ▸).

## 3. Supra­molecular features

In the crystal, the N1*A*—H1*A*⋯O1*B* and N2*A*—H2*AB*⋯O2*B* hydrogen bonds (Table 1 ▸) link the cation and the anion of the asymmetric unit into an 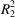
(8) graph-set motif (Etter *et al.*, 1990 ▸), in which the carboxyl­ate group bridges the two N—H donor groups of the cation (Fig. 1 ▸). The third hydrogen bond, N2*A*—H2*AA*⋯O1*B*^i^, connects these ion pairs into layers parallel to (100), Fig. 2 ▸.

Within these layers, the carboxyl­ate atom O1*B* is located above the benzo­thia­zolium ring system of a cation related by translation along the *b* axis, with O1*B*⋯*Cg* distances of 3.486 (4), 3.711 (4) and 3.440 (4) Å to the centroids of the thia­zolium ring, the phenyl ring and the entire nine-membered ring system, respectively. Neither significant π–π stacking inter­actions [shortest centroid-to-centroid separation is 4.557 (4) Å] nor C—H⋯π inter­actions are observed.

## 4. Hirshfeld surface analysis

A Hirshfeld surface analysis was performed with *CrystalExplorer* (Spackman *et al.*, 2021 ▸) to qu­antify inter­molecular contacts in the crystal packing. The cation (*A*) and the anion (*B*) were selected separately, which allows the donor/acceptor environments of the ions to be compared directly with the hydrogen-bonding scheme. The percentage contributions of the individual contacts are summarized in Fig. 3 ▸, and the corresponding two-dimensional fingerprint plots are shown in Fig. 4 ▸.

For cation *A*, the Hirshfeld surface volume and area are 195.33 Å^3^ and 194.85 Å^2^, with a globularity of 0.836 and an asphericity of 0.195 (Fig. 5 ▸*a*). H⋯H contacts make the largest contribution, followed by C⋯H/H⋯C, O⋯H/H⋯O and S⋯H/H⋯S contacts (Figs. 3 ▸, 4 ▸). Because the cation contains no O atoms, its O⋯H/H⋯O contribution necessarily arises from cationic H atoms in contact with O atoms of neighbouring anions, in agreement with the three N—H⋯O hydrogen bonds, which appear as the most intense red spots on the *d*_norm_ surface (Fig. 5 ▸*a*).

For anion *B*, the volume and area increase to 255.79 Å^3^ and 248.36 Å^2^, while the globularity decreases to 0.785 and the asphericity increases slightly to 0.207 (Fig. 5 ▸*b*). Its fingerprint plot is more heterogeneous, with H⋯H and S⋯H/H⋯S contacts making the largest contributions, followed by C⋯H/H⋯C and O⋯H/H⋯O contacts (Figs. 3 ▸, 4 ▸). The O_inside_⋯H_outside_ contribution of the anion closely matches the complementary H_inside_⋯O_outside_ contribution of the cation; the two independently calculated surfaces therefore describe opposite sides of the same N—H⋯O contacts.

The larger surface, lower globularity and higher asphericity of the anion are consistent with its additional propano­ate chain, and its sulfur-rich surface accounts for the much larger S⋯H/H⋯S contribution compared with the cation (Fig. 3 ▸). Overall, the Hirshfeld analysis shows that charge-assisted N—H⋯O hydrogen bonds are the principal directional inter­actions, supplemented by H⋯H, C⋯H/H⋯C and S⋯H/H⋯S contacts. The percentages represent fractions of the Hirshfeld surface area and therefore indicate contact prevalence rather than inter­action strength.

## 5. Database survey

A search of the Cambridge Structural Database (CSD, Version 6.01, November 2025, including the February 2026 updates; Groom *et al.*, 2016 ▸) revealed no structure containing the title ion pair. The discussion below is therefore restricted to the most closely related systems, *i.e.* salts of 2-amino­benzo­thia­zoles with carb­oxy­lic acids and compounds containing the (1,3-benzo­thia­zol-2-ylsulfan­yl)alkanoate fragment.

The structure of the neutral base, 6-methyl-1,3-benzo­thia­zol-2-amine, has been reported by Saeed *et al.* (2007 ▸). Protonation of 2-amino­benzo­thia­zoles by carb­oxy­lic acids at the endocyclic N atom is well documented. In the series of 2-amino­benzo­thia­zole adducts with carb­oxy­lic acids described by Lynch *et al.* (1998 ▸, 1999 ▸), a frequently observed association between the base and the acid is the 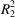
(8) graph-set motif formed by the ring N—H and amino N—H donors and the two O atoms of a carb­oxy(late) group, *i.e.* the same heterosynthon as found in the title salt. The same cation as in the title compound, 6-methyl-2,3-di­hydro-1,3-benzo­thia­zol-2-iminium, has been characterized as a salt with dipicolinic acid (Ilkimen *et al.*, 2013 ▸), and further examples of 2-amino­benzo­thia­zolium carboxyl­ates have been described by Zhang *et al.* (2009 ▸), Chen *et al.* (2020 ▸), Gao *et al.* (2021 ▸) and Tsemeugne *et al.* (2025 ▸).

The anion of the title salt is the higher homologue of 2-(1,3-benzo­thia­zol-2-ylsulfan­yl)acetate, which has been structurally characterized as the free acid (Fang & Wang, 2010 ▸) and as a ligand in Cu^II^ and Cd^II^ complexes (Bian *et al.*, 2007*a* ▸,*b* ▸). Closely related benzo­thia­zole-derived carboxyl­ate salts with amines have also been reported by our group (Ashurov *et al.*, 2016 ▸, 2017 ▸, 2018 ▸; Ashurov, 2016 ▸). Compared with the acetate analogues, the additional methyl­ene group of the propano­ate chain increases its conformational freedom, which in the title salt allows the carboxyl­ate group to fold away from the benzo­thia­zole plane and to face the donor groups of the cation.

## 6. Synthesis and crystallization

All starting materials and solvents were obtained from commercial sources and used without further purification. 6-Methyl-1,3-benzo­thia­zol-2-amine (40 mg, 0.244 mmol) and 3-(1,3-benzo­thia­zol-2-ylsulfan­yl)propanoic acid (58 mg, 0.242 mmol) were mixed in a 1:1 molar ratio and dissolved in a 1:1 (*v*/*v*) mixture of methanol and ethanol. The mixture was treated in an ultrasonic bath operating at 30 kHz and maintained at 333 K until a clear solution was obtained. The resulting solution was then allowed to crystallize at 293 K by slow evaporation. After approximately 3 d, yellow block-shaped single crystals suitable for X-ray diffraction were obtained.

## 7. Refinement

Crystal data, data collection and structure refinement details are summarized in Table 2 ▸. H atoms bonded to C atoms were placed in calculated positions and refined using a riding model, with C—H = 0.93 Å (aromatic), 0.96 Å (meth­yl) and 0.97 Å (methyl­ene), and with *U*_iso_(H) = 1.2*U*_eq_(C) for aromatic and methyl­ene H atoms and 1.5*U*_eq_(C) for methyl H atoms. The three N-bound H atoms were located in difference-Fourier maps and refined with restrained N—H distances of 0.86 (1) Å.

## Supplementary Material

Crystal structure: contains datablock(s) I. DOI: 10.1107/S2056989026010686/wm5815sup1.cif

Structure factors: contains datablock(s) I. DOI: 10.1107/S2056989026010686/wm5815Isup2.hkl

Supporting information file. DOI: 10.1107/S2056989026010686/wm5815Isup3.cml

CCDC reference: 2515049

Additional supporting information:  crystallographic information; 3D view; checkCIF report

## Figures and Tables

**Figure 1:**
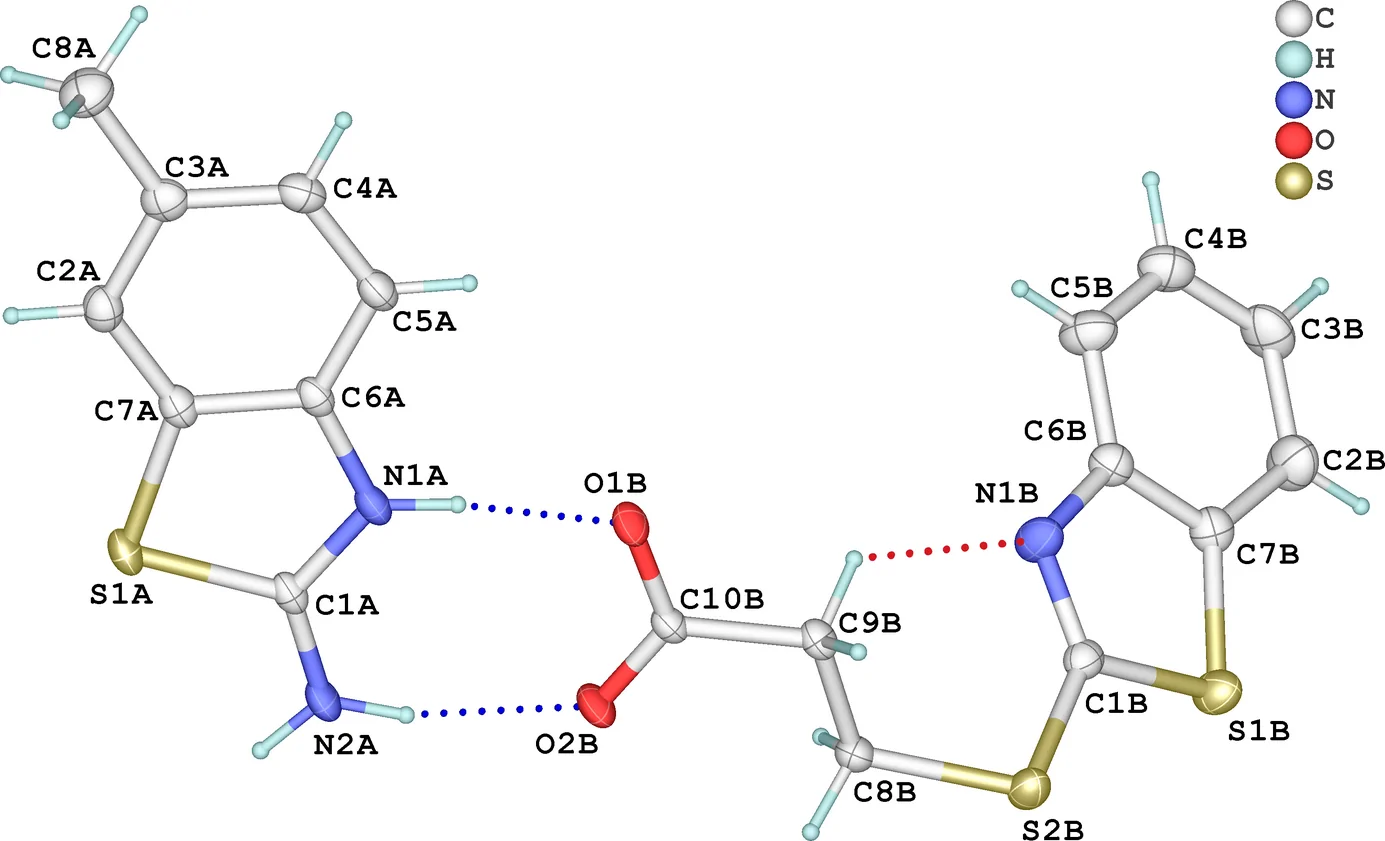
The asymmetric unit of the title compound, showing the atom-numbering scheme; displacement ellipsoids are drawn at the 50% probability level and H atoms are shown as small spheres of arbitrary radius. N—H⋯O hydrogen bonds forming the 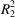
(8) graph-set motif are shown as blue dashed lines and the intra­molecular C9*B*—H9*BA*⋯N1*B* contact as a red dashed line.

**Figure 2:**
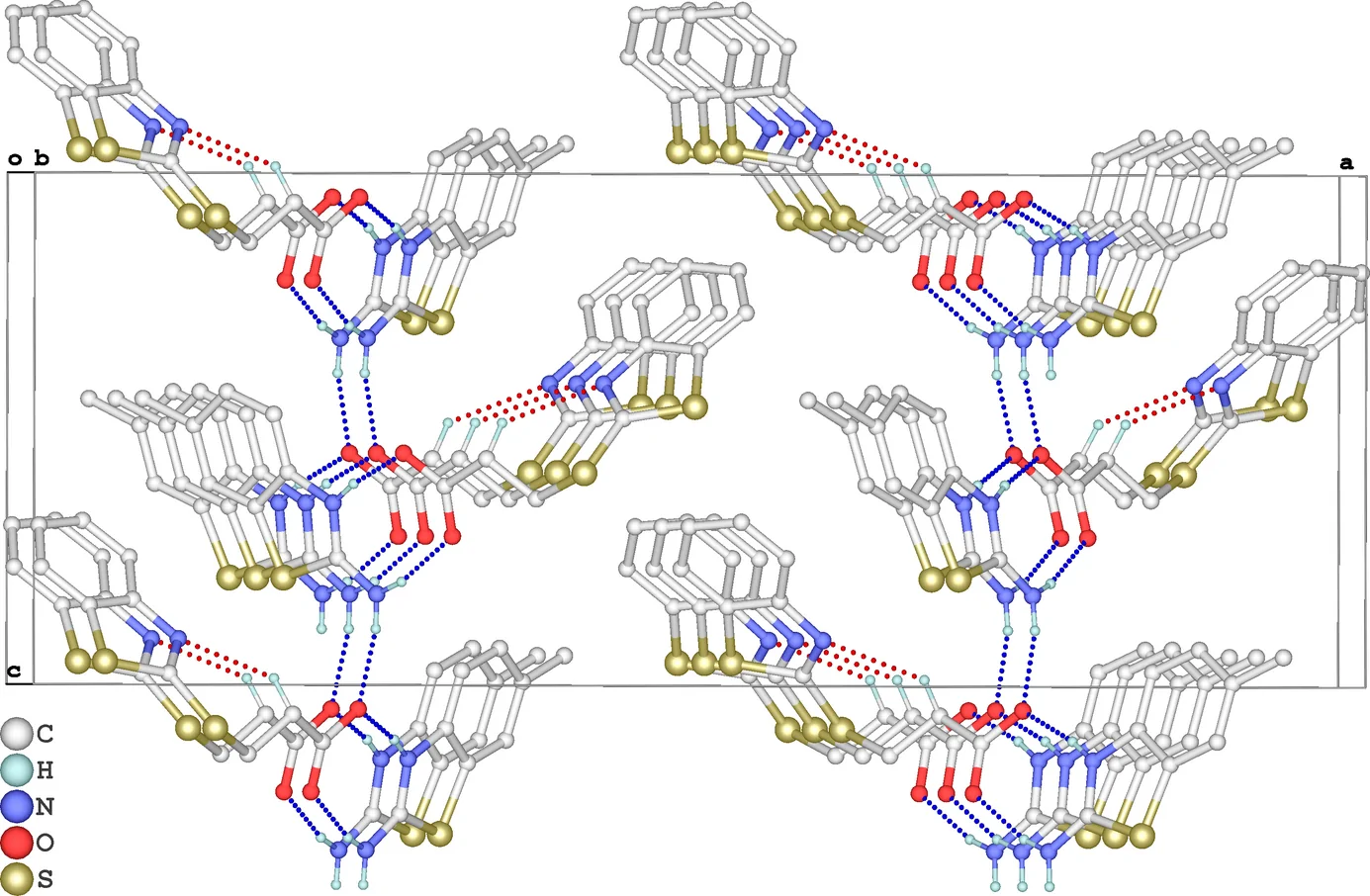
Part of the crystal structure viewed approximately along the *b* axis, showing the N—H⋯O hydrogen-bonded layer extending parallel to (100). N—H⋯O hydrogen bonds are shown as blue and C—H⋯N contacts as red dashed lines; H atoms not involved in hydrogen bonding have been omitted for clarity.

**Figure 3:**
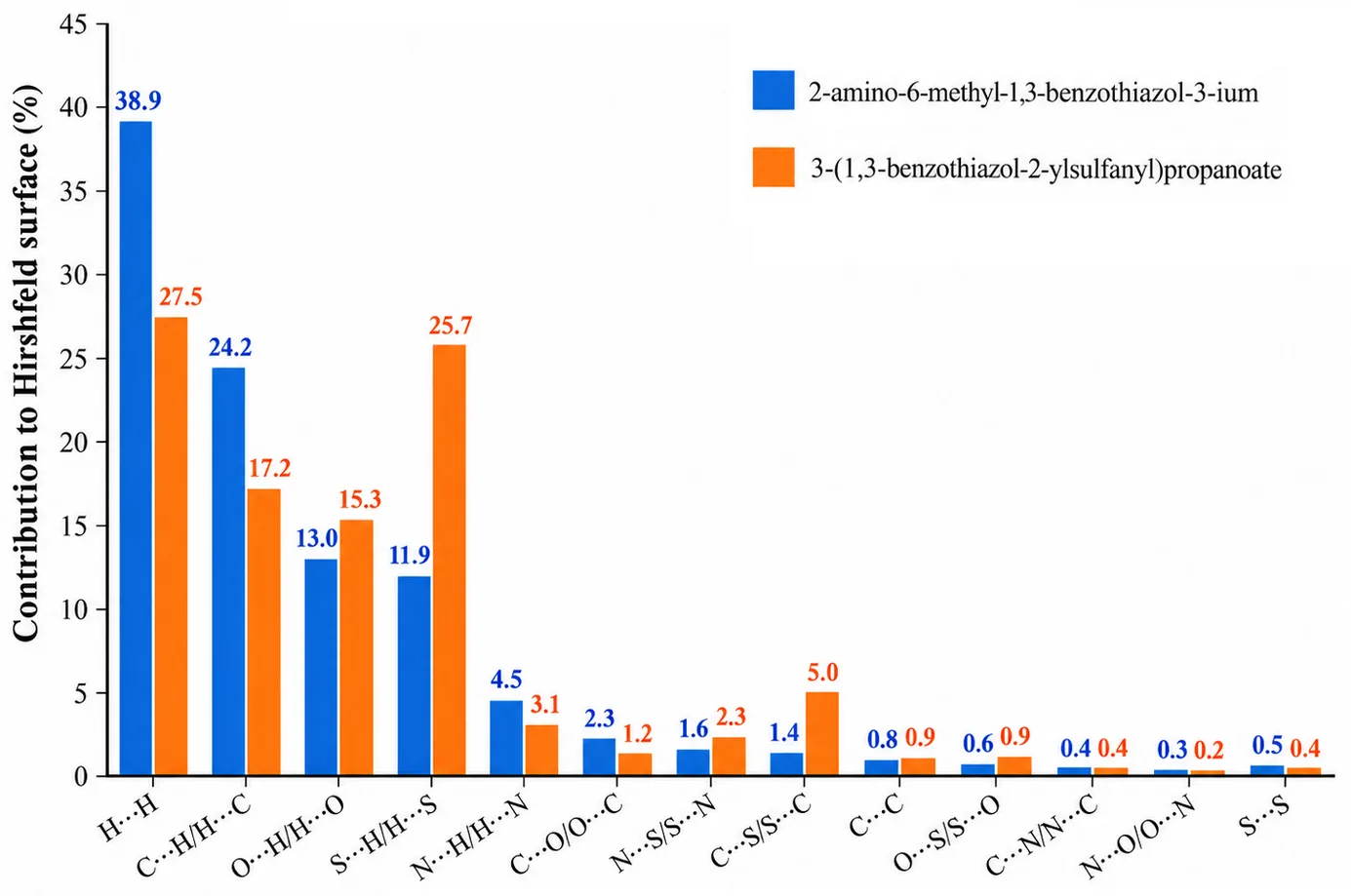
Percentage contributions of inter­molecular contacts to the Hirshfeld surfaces of the 6-methyl-2,3-di­hydro-1,3-benzo­thia­zol-2-iminium cation (*A*) and the 3-(1,3-benzo­thia­zol-2-ylsulfan­yl)propano­ate anion (*B*).

**Figure 4:**
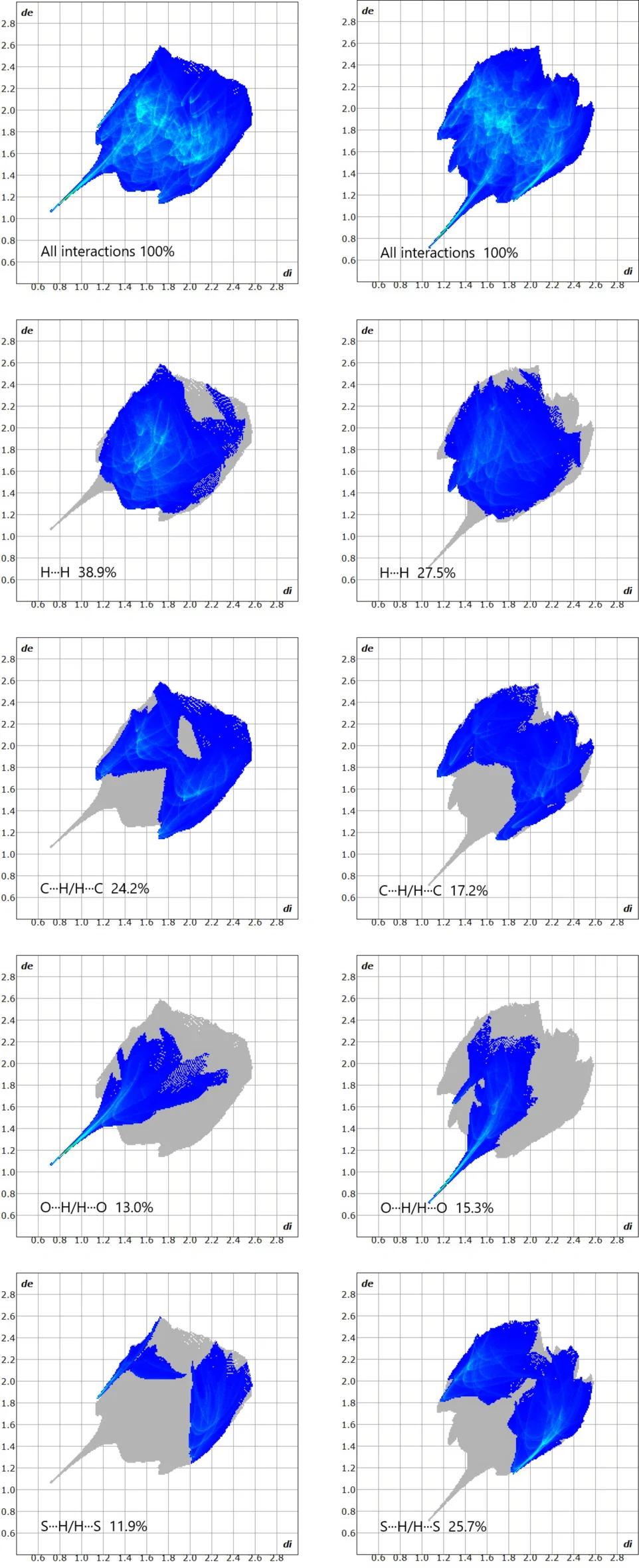
Two-dimensional fingerprint plots for the cation (left) and anion (right): full, and resolved into H⋯H, C⋯H/H⋯C, O⋯H/H⋯O and S⋯H/H⋯S contacts.

**Figure 5:**
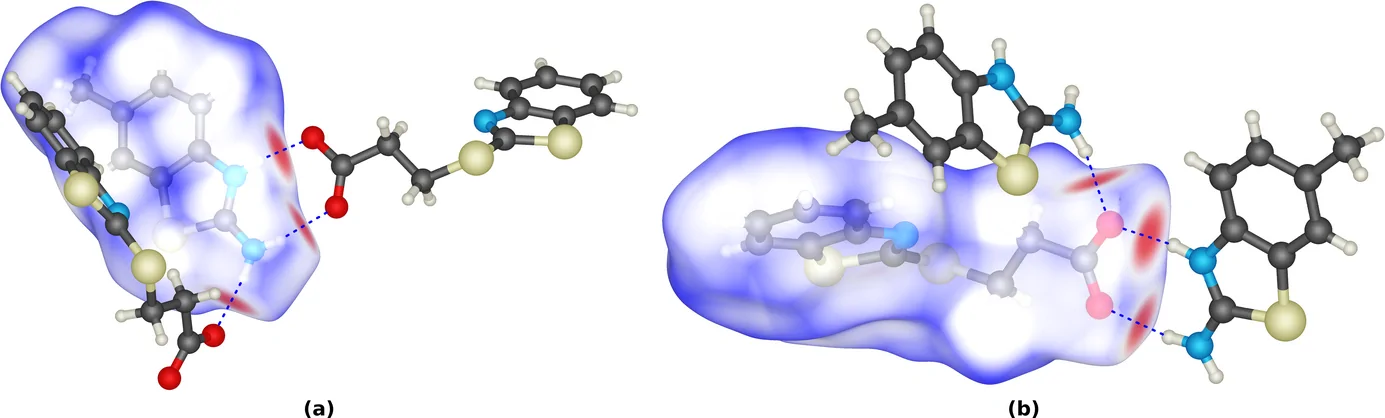
Hirshfeld surfaces mapped over *d*_norm_ for (*a*) the cation and (*b*) the anion. Red spots mark close inter­molecular contacts corresponding to the N—H⋯O hydrogen bonds.

**Table 1:** Hydrogen-bond geometry (Å, °)

*D*—H⋯*A*	*D*—H	H⋯*A*	*D*⋯*A*	*D*—H⋯*A*
N1*A*—H1*A*⋯O1*B*	0.86 (1)	1.78 (2)	2.633 (5)	171 (8)
N2*A*—H2*AA*⋯O1*B*^i^	0.86 (1)	1.94 (2)	2.785 (5)	167 (4)
N2*A*—H2*AB*⋯O2*B*	0.86 (1)	1.95 (2)	2.789 (6)	166 (6)
C9*B*—H9*BA*⋯N1*B*	0.97	2.52	3.197 (7)	127

Symmetry code: (i) 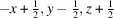.

**Table 2:** Experimental details

Crystal data	
Chemical formula	C_8_H_9_N_2_S^+^·C_10_H_8_NO_2_S_2_^−^
*M*_r_	403.52
Crystal system, space group	Orthorhombic, *Pna*2_1_
Temperature (K)	292
*a*, *b*, *c* (Å)	30.7964 (15), 5.1070 (2), 11.7505 (5)
*V* (Å^3^)	1848.09 (14)
*Z*	4
Radiation type	Cu *K*α
μ (mm^−1^)	3.82
Crystal size (mm)	0.44 × 0.30 × 0.12

Data collection	
Diffractometer	Xcalibur, Ruby
Absorption correction	Multi-scan (*CrysAlis PRO*; Rigaku OD, 2023 ▸)
*T*_min_, *T*_max_	0.493, 1.000
No. of measured, independent and observed [*I* > 2σ(*I*)] reflections	11887, 3443, 2823
*R*_int_	0.050
(sin θ/λ)_max_ (Å^−1^)	0.632

Refinement	
*R*[*F*^2^ > 2σ(*F*^2^)], *wR*(*F*^2^), *S*	0.043, 0.110, 1.05
No. of reflections	3443
No. of parameters	249
No. of restraints	4
H-atom treatment	H atoms treated by a mixture of independent and constrained refinement
Δρ_max_, Δρ_min_ (e Å^−3^)	0.24, −0.29
Absolute structure	Flack *x* determined using 944 quotients [(*I*^+^)−(*I*^−^)]/[(*I*^+^)+(*I*^−^)] (Parsons *et al.*, 2013 ▸)
Absolute structure parameter	0.041 (14)

Computer programs: *CrysAlis PRO* (Rigaku OD, 2023 ▸), *SHELXT* (Sheldrick, 2015*a* ▸), *SHELXL* (Sheldrick, 2015*b* ▸), *OLEX2* (Dolomanov *et al.*, 2009 ▸) and *publCIF* (Westrip, 2010 ▸).

## supplementary crystallographic information

### 6-Methyl-2,3-dihydro-1,3-benzothiazol-2-iminium 3-(1,3-benzothiazol-2-ylsulfanyl)propanoate Crystal data

**Table:**

C_8_H_9_N_2_S^+^·C_10_H_8_NO_2_S_2_^−^	*D*_x_ = 1.450 Mg m^−^^3^
*M**_r_* = 403.52	Cu *K*α radiation, λ = 1.54184 Å
Orthorhombic, *P**n**a*2_1_	Cell parameters from 3206 reflections
*a* = 30.7964 (15) Å	θ = 4.7–75.1°
*b* = 5.1070 (2) Å	µ = 3.82 mm^−^^1^
*c* = 11.7505 (5) Å	*T* = 292 K
*V* = 1848.09 (14) Å^3^	Block, yellow
*Z* = 4	0.44 × 0.30 × 0.12 mm
*F*(000) = 840	

### 6-Methyl-2,3-dihydro-1,3-benzothiazol-2-iminium 3-(1,3-benzothiazol-2-ylsulfanyl)propanoate Data collection

**Table:**

Xcalibur, Ruby diffractometer	3443 independent reflections
Radiation source: fine-focus sealed X-ray tube, Enhance (Cu) X-ray Source	2823 reflections with *I* > 2σ(*I*)
Graphite monochromator	*R*_int_ = 0.050
Detector resolution: 10.2576 pixels mm^-1^	θ_max_ = 77.2°, θ_min_ = 4.7°
ω scans	*h* = −32→38
Absorption correction: multi-scan (CrysAlisPRO; Rigaku OD, 2023)	*k* = −4→6
*T*_min_ = 0.493, *T*_max_ = 1.000	*l* = −12→14
11887 measured reflections	

### 6-Methyl-2,3-dihydro-1,3-benzothiazol-2-iminium 3-(1,3-benzothiazol-2-ylsulfanyl)propanoate Refinement

**Table:**

Refinement on *F*^2^	H atoms treated by a mixture of independent and constrained refinement
Least-squares matrix: full	*w* = 1/[σ^2^(*F*_o_^2^) + (0.051*P*)^2^ + 0.2766*P*] where *P* = (*F*_o_^2^ + 2*F*_c_^2^)/3
*R*[*F*^2^ > 2σ(*F*^2^)] = 0.043	(Δ/σ)_max_ < 0.001
*wR*(*F*^2^) = 0.110	Δρ_max_ = 0.24 e Å^−^^3^
*S* = 1.05	Δρ_min_ = −0.29 e Å^−^^3^
3443 reflections	Extinction correction: SHELXL (Sheldrick, 2015b), Fc^*^=kFc[1+0.001xFc^2^λ^3^/sin(2θ)]^-1/4^
249 parameters	Extinction coefficient: 0.0018 (3)
4 restraints	Absolute structure: Flack *x* determined using 944 quotients [(*I*^+^)-(*I*^-^)]/[(*I*^+^)+(*I*^-^)] (Parsons *et al.*, 2013)
Primary atom site location: dual	Absolute structure parameter: 0.041 (14)
Hydrogen site location: mixed	

### 6-Methyl-2,3-dihydro-1,3-benzothiazol-2-iminium 3-(1,3-benzothiazol-2-ylsulfanyl)propanoate Special details

**Table:**

Geometry. All esds (except the esd in the dihedral angle between two l.s. planes) are estimated using the full covariance matrix. The cell esds are taken into account individually in the estimation of esds in distances, angles and torsion angles; correlations between esds in cell parameters are only used when they are defined by crystal symmetry. An approximate (isotropic) treatment of cell esds is used for estimating esds involving l.s. planes.

### 6-Methyl-2,3-dihydro-1,3-benzothiazol-2-iminium 3-(1,3-benzothiazol-2-ylsulfanyl)propanoate Fractional atomic coordinates and isotropic or equivalent isotropic displacement parameters (Å^2^)

**Table:**

	*x*	*y*	*z*	*U*_iso_*/*U*_eq_	
S1A	0.18588 (4)	0.0760 (3)	0.79054 (11)	0.0589 (4)	
N1A	0.21646 (15)	0.3990 (8)	0.6445 (3)	0.0508 (10)	
H1A	0.229 (2)	0.521 (10)	0.607 (6)	0.10 (3)*	
N2A	0.24873 (16)	0.4349 (9)	0.8222 (3)	0.0557 (11)	
H2AA	0.2493 (13)	0.373 (8)	0.8903 (18)	0.038 (12)*	
H2AB	0.2648 (15)	0.561 (7)	0.798 (5)	0.063 (16)*	
C1A	0.22130 (16)	0.3295 (9)	0.7516 (4)	0.0475 (11)	
C2A	0.13320 (18)	−0.0924 (10)	0.6066 (5)	0.0606 (13)	
H2A	0.119773	−0.217258	0.652179	0.073*	
C3A	0.12133 (18)	−0.0660 (11)	0.4931 (5)	0.0623 (14)	
C4A	0.14236 (18)	0.1227 (12)	0.4277 (5)	0.0633 (14)	
H4A	0.134734	0.140290	0.351531	0.076*	
C5A	0.17408 (18)	0.2845 (10)	0.4716 (5)	0.0590 (13)	
H5A	0.187478	0.409818	0.426178	0.071*	
C6A	0.18543 (15)	0.2564 (9)	0.5841 (4)	0.0485 (10)	
C7A	0.16520 (17)	0.0685 (10)	0.6515 (4)	0.0524 (12)	
C8A	0.0865 (2)	−0.2352 (15)	0.4416 (7)	0.0838 (19)	
H8AA	0.089499	−0.411306	0.469066	0.126*	
H8AB	0.089258	−0.233901	0.360191	0.126*	
H8AC	0.058465	−0.168444	0.462547	0.126*	
S1B	0.46617 (5)	1.4758 (3)	0.45498 (15)	0.0733 (5)	
S2B	0.38322 (5)	1.4749 (3)	0.58497 (13)	0.0642 (4)	
O1B	0.26169 (11)	0.7748 (7)	0.5471 (3)	0.0561 (9)	
O2B	0.29775 (12)	0.8130 (8)	0.7089 (3)	0.0674 (10)	
N1B	0.40442 (16)	1.1455 (10)	0.4106 (5)	0.0714 (13)	
C1B	0.41524 (16)	1.3358 (10)	0.4781 (5)	0.0530 (11)	
C2B	0.5093 (2)	1.2283 (15)	0.2708 (7)	0.092 (2)	
H2B	0.533518	1.335769	0.277052	0.111*	
C3B	0.5071 (3)	1.0375 (16)	0.1891 (7)	0.092 (2)	
H3B	0.530234	1.015593	0.139055	0.111*	
C4B	0.4713 (2)	0.8777 (15)	0.1800 (6)	0.0824 (19)	
H4B	0.470687	0.748542	0.124267	0.099*	
C5B	0.4368 (2)	0.9054 (14)	0.2511 (6)	0.084 (2)	
H5B	0.412742	0.796525	0.243999	0.101*	
C6B	0.43783 (18)	1.0970 (11)	0.3341 (5)	0.0585 (13)	
C7B	0.47380 (18)	1.2572 (11)	0.3451 (5)	0.0615 (13)	
C8B	0.35287 (16)	1.1948 (10)	0.6354 (4)	0.0539 (12)	
H8BA	0.373046	1.052014	0.648443	0.065*	
H8BB	0.339972	1.239302	0.708223	0.065*	
C9B	0.31742 (16)	1.0987 (9)	0.5576 (4)	0.0495 (11)	
H9BA	0.330319	1.035694	0.487430	0.059*	
H9BB	0.298403	1.243736	0.538750	0.059*	
C10B	0.29060 (15)	0.8794 (9)	0.6110 (4)	0.0451 (10)	

### 6-Methyl-2,3-dihydro-1,3-benzothiazol-2-iminium 3-(1,3-benzothiazol-2-ylsulfanyl)propanoate Atomic displacement parameters (Å^2^)

**Table:**

	*U*^11^	*U*^22^	*U*^33^	*U*^12^	*U*^13^	*U*^23^
S1A	0.0684 (8)	0.0628 (7)	0.0454 (6)	−0.0024 (6)	0.0089 (6)	0.0176 (6)
N1A	0.061 (2)	0.055 (2)	0.036 (2)	0.004 (2)	0.0096 (18)	0.0088 (18)
N2A	0.068 (3)	0.061 (3)	0.038 (2)	−0.003 (2)	0.004 (2)	0.015 (2)
C1A	0.052 (3)	0.050 (2)	0.041 (2)	0.010 (2)	0.011 (2)	0.010 (2)
C2A	0.061 (3)	0.057 (3)	0.064 (4)	0.002 (2)	0.013 (3)	0.005 (3)
C3A	0.059 (3)	0.064 (3)	0.064 (4)	0.008 (3)	0.008 (3)	−0.010 (3)
C4A	0.072 (4)	0.068 (4)	0.050 (3)	0.009 (3)	0.000 (3)	−0.007 (3)
C5A	0.074 (4)	0.060 (3)	0.042 (3)	0.005 (3)	0.008 (3)	0.006 (2)
C6A	0.053 (3)	0.052 (2)	0.041 (2)	0.008 (2)	0.012 (2)	0.003 (2)
C7A	0.055 (3)	0.054 (3)	0.048 (3)	0.008 (2)	0.007 (2)	0.008 (2)
C8A	0.072 (4)	0.087 (4)	0.092 (5)	−0.007 (3)	−0.008 (4)	−0.010 (4)
S1B	0.0636 (9)	0.0799 (10)	0.0766 (10)	−0.0135 (7)	0.0061 (7)	−0.0154 (8)
S2B	0.0711 (9)	0.0545 (7)	0.0669 (9)	−0.0085 (6)	0.0140 (7)	−0.0119 (7)
O1B	0.060 (2)	0.065 (2)	0.0430 (18)	−0.0083 (17)	−0.0037 (15)	0.0166 (17)
O2B	0.081 (3)	0.084 (3)	0.0366 (18)	−0.020 (2)	−0.0037 (18)	0.0164 (19)
N1B	0.065 (3)	0.068 (3)	0.081 (3)	−0.005 (2)	0.017 (2)	−0.018 (3)
C1B	0.056 (3)	0.050 (3)	0.053 (3)	−0.002 (2)	−0.005 (2)	0.001 (2)
C2B	0.077 (4)	0.095 (5)	0.106 (6)	−0.009 (4)	0.029 (4)	−0.011 (4)
C3B	0.096 (6)	0.095 (5)	0.085 (5)	0.024 (4)	0.036 (4)	−0.002 (4)
C4B	0.089 (5)	0.083 (5)	0.076 (4)	0.016 (4)	0.003 (4)	−0.015 (4)
C5B	0.073 (4)	0.089 (5)	0.090 (5)	−0.001 (3)	0.005 (3)	−0.030 (4)
C6B	0.057 (3)	0.058 (3)	0.060 (3)	0.008 (2)	0.001 (2)	−0.001 (3)
C7B	0.058 (3)	0.060 (3)	0.067 (3)	0.008 (2)	0.003 (3)	0.004 (3)
C8B	0.067 (3)	0.053 (3)	0.042 (3)	−0.006 (2)	0.001 (2)	−0.002 (2)
C9B	0.052 (3)	0.054 (3)	0.042 (3)	0.003 (2)	0.002 (2)	0.008 (2)
C10B	0.052 (3)	0.049 (2)	0.035 (2)	0.0048 (19)	0.0041 (19)	0.0041 (19)

### 6-Methyl-2,3-dihydro-1,3-benzothiazol-2-iminium 3-(1,3-benzothiazol-2-ylsulfanyl)propanoate Geometric parameters (Å, º)

**Table:**

S1A—C1A	1.753 (5)	S2B—C1B	1.747 (6)
S1A—C7A	1.754 (5)	S2B—C8B	1.809 (5)
N1A—H1A	0.861 (14)	O1B—C10B	1.281 (6)
N1A—C1A	1.316 (6)	O2B—C10B	1.220 (6)
N1A—C6A	1.396 (7)	N1B—C1B	1.298 (7)
N2A—H2AA	0.862 (13)	N1B—C6B	1.389 (7)
N2A—H2AB	0.857 (14)	C2B—H2B	0.9300
N2A—C1A	1.300 (7)	C2B—C3B	1.369 (10)
C2A—H2A	0.9300	C2B—C7B	1.405 (8)
C2A—C3A	1.389 (8)	C3B—H3B	0.9300
C2A—C7A	1.388 (8)	C3B—C4B	1.377 (10)
C3A—C4A	1.392 (8)	C4B—H4B	0.9300
C3A—C8A	1.505 (9)	C4B—C5B	1.359 (9)
C4A—H4A	0.9300	C5B—H5B	0.9300
C4A—C5A	1.380 (8)	C5B—C6B	1.381 (8)
C5A—H5A	0.9300	C6B—C7B	1.383 (8)
C5A—C6A	1.374 (7)	C8B—H8BA	0.9700
C6A—C7A	1.392 (7)	C8B—H8BB	0.9700
C8A—H8AA	0.9600	C8B—C9B	1.506 (7)
C8A—H8AB	0.9600	C9B—H9BA	0.9700
C8A—H8AC	0.9600	C9B—H9BB	0.9700
S1B—C1B	1.745 (5)	C9B—C10B	1.526 (6)
S1B—C7B	1.723 (6)		
			
C1A—S1A—C7A	89.9 (2)	S1B—C1B—S2B	116.9 (3)
C1A—N1A—H1A	129 (5)	N1B—C1B—S1B	116.2 (4)
C1A—N1A—C6A	115.0 (4)	N1B—C1B—S2B	126.8 (4)
C6A—N1A—H1A	116 (5)	C3B—C2B—H2B	120.9
H2AA—N2A—H2AB	125 (5)	C3B—C2B—C7B	118.2 (7)
C1A—N2A—H2AA	117 (3)	C7B—C2B—H2B	120.9
C1A—N2A—H2AB	118 (4)	C2B—C3B—H3B	119.5
N1A—C1A—S1A	112.2 (4)	C2B—C3B—C4B	121.0 (6)
N2A—C1A—S1A	122.9 (4)	C4B—C3B—H3B	119.5
N2A—C1A—N1A	124.8 (5)	C3B—C4B—H4B	119.4
C3A—C2A—H2A	120.2	C5B—C4B—C3B	121.2 (7)
C7A—C2A—H2A	120.2	C5B—C4B—H4B	119.4
C7A—C2A—C3A	119.7 (5)	C4B—C5B—H5B	120.4
C2A—C3A—C4A	118.3 (5)	C4B—C5B—C6B	119.3 (6)
C2A—C3A—C8A	121.2 (6)	C6B—C5B—H5B	120.4
C4A—C3A—C8A	120.5 (6)	C5B—C6B—N1B	124.5 (6)
C3A—C4A—H4A	118.7	C5B—C6B—C7B	120.2 (5)
C5A—C4A—C3A	122.5 (5)	C7B—C6B—N1B	115.3 (5)
C5A—C4A—H4A	118.7	C2B—C7B—S1B	129.7 (5)
C4A—C5A—H5A	120.8	C6B—C7B—S1B	110.1 (4)
C6A—C5A—C4A	118.5 (5)	C6B—C7B—C2B	120.1 (6)
C6A—C5A—H5A	120.8	S2B—C8B—H8BA	108.4
C5A—C6A—N1A	127.5 (4)	S2B—C8B—H8BB	108.4
C5A—C6A—C7A	120.4 (5)	H8BA—C8B—H8BB	107.4
C7A—C6A—N1A	112.1 (4)	C9B—C8B—S2B	115.7 (3)
C2A—C7A—S1A	128.7 (4)	C9B—C8B—H8BA	108.4
C2A—C7A—C6A	120.6 (5)	C9B—C8B—H8BB	108.4
C6A—C7A—S1A	110.7 (4)	C8B—C9B—H9BA	109.1
C3A—C8A—H8AA	109.5	C8B—C9B—H9BB	109.1
C3A—C8A—H8AB	109.5	C8B—C9B—C10B	112.4 (4)
C3A—C8A—H8AC	109.5	H9BA—C9B—H9BB	107.8
H8AA—C8A—H8AB	109.5	C10B—C9B—H9BA	109.1
H8AA—C8A—H8AC	109.5	C10B—C9B—H9BB	109.1
H8AB—C8A—H8AC	109.5	O1B—C10B—C9B	116.2 (4)
C7B—S1B—C1B	88.5 (3)	O2B—C10B—O1B	124.2 (4)
C1B—S2B—C8B	101.9 (2)	O2B—C10B—C9B	119.6 (4)
C1B—N1B—C6B	109.8 (5)		
			
N1A—C6A—C7A—S1A	0.0 (5)	N1B—C6B—C7B—C2B	−179.0 (6)
N1A—C6A—C7A—C2A	179.7 (4)	C1B—S1B—C7B—C2B	179.2 (7)
C1A—S1A—C7A—C2A	179.7 (5)	C1B—S1B—C7B—C6B	0.0 (4)
C1A—S1A—C7A—C6A	−0.6 (4)	C1B—S2B—C8B—C9B	−73.6 (4)
C1A—N1A—C6A—C5A	−179.2 (5)	C1B—N1B—C6B—C5B	179.5 (6)
C1A—N1A—C6A—C7A	0.8 (6)	C1B—N1B—C6B—C7B	−0.6 (7)
C2A—C3A—C4A—C5A	−0.6 (8)	C2B—C3B—C4B—C5B	0.5 (12)
C3A—C2A—C7A—S1A	179.8 (4)	C3B—C2B—C7B—S1B	−179.6 (6)
C3A—C2A—C7A—C6A	0.1 (8)	C3B—C2B—C7B—C6B	−0.5 (10)
C3A—C4A—C5A—C6A	0.5 (8)	C3B—C4B—C5B—C6B	−0.2 (11)
C4A—C5A—C6A—N1A	180.0 (5)	C4B—C5B—C6B—N1B	179.3 (6)
C4A—C5A—C6A—C7A	0.0 (7)	C4B—C5B—C6B—C7B	−0.5 (10)
C5A—C6A—C7A—S1A	−180.0 (4)	C5B—C6B—C7B—S1B	−179.8 (5)
C5A—C6A—C7A—C2A	−0.2 (7)	C5B—C6B—C7B—C2B	0.9 (9)
C6A—N1A—C1A—S1A	−1.3 (5)	C6B—N1B—C1B—S1B	0.7 (6)
C6A—N1A—C1A—N2A	179.2 (5)	C6B—N1B—C1B—S2B	177.5 (4)
C7A—S1A—C1A—N1A	1.0 (4)	C7B—S1B—C1B—S2B	−177.6 (3)
C7A—S1A—C1A—N2A	−179.4 (4)	C7B—S1B—C1B—N1B	−0.4 (5)
C7A—C2A—C3A—C4A	0.2 (8)	C7B—C2B—C3B—C4B	−0.2 (12)
C7A—C2A—C3A—C8A	−179.4 (5)	C8B—S2B—C1B—S1B	−148.8 (3)
C8A—C3A—C4A—C5A	179.1 (5)	C8B—S2B—C1B—N1B	34.3 (6)
S2B—C8B—C9B—C10B	−174.5 (3)	C8B—C9B—C10B—O1B	−175.4 (4)
N1B—C6B—C7B—S1B	0.3 (7)	C8B—C9B—C10B—O2B	4.1 (6)

### 6-Methyl-2,3-dihydro-1,3-benzothiazol-2-iminium 3-(1,3-benzothiazol-2-ylsulfanyl)propanoate Hydrogen-bond geometry (Å, º)

**Table:**

*D*—H···*A*	*D*—H	H···*A*	*D*···*A*	*D*—H···*A*
N1*A*—H1*A*···O1*B*	0.86 (1)	1.78 (2)	2.633 (5)	171 (8)
N2*A*—H2*AA*···O1*B*^i^	0.86 (1)	1.94 (2)	2.785 (5)	167 (4)
N2*A*—H2*AB*···O2*B*	0.86 (1)	1.95 (2)	2.789 (6)	166 (6)
C9*B*—H9*BA*···N1*B*	0.97	2.52	3.197 (7)	127

Symmetry code: (i) −*x*+1/2, *y*−1/2, *z*+1/2.
